# Zolpidem reduces pain intensity postoperatively: a systematic review and meta-analysis of the effect of hypnotic medicines on post-operative pain intensity

**DOI:** 10.1186/s13643-020-01458-8

**Published:** 2020-09-03

**Authors:** Edel T. O’Hagan, Markus Hübscher, Christopher B. Miller, Christopher J. Gordon, Sylvia Gustin, Nancy Briggs, James H. McAuley

**Affiliations:** 1 Centre for Pain IMPACT, Neuroscience Research Australia, Randwick, NSW 2031 Australia; 2 Prince of Wales Clinical School, The University of New South Wales, Randwick, NSW 2031 Australia; 3 CIRUS, Centre for Sleep and Chronobiology, Woolcock Institute of Medical Research, The University of Sydney, Glebe, NSW 2037 Australia; 4 Faculty of Medicine and Health, The University of Sydney, Camperdown, NSW 2050 Australia; 5 Gustin Pain Imaging Laboratory, School of Psychology, The University of New South Wales, Randwick, NSW 2031 Australia; 6 Stats Central, University of New South Wales, Randwick, NSW 2031 Australia; 7 School of Medical Science, University of New South Wales, Randwick, NSW 2031 Australia

## Abstract

**Background:**

This systematic review aimed to investigate whether the administration of hypnotic medicines, z-drugs, melatonin or benzodiazepines, reduced pain intensity postoperatively.

**Methods:**

Medline, Embase, Cinahl, Psych info, Central and PubMed databases were searched, from inception to February 2020 to identify relevant trials. The search was extended, post hoc, to include meta-Register of Controlled Trials, the Web of Science and the conference booklets for the 14th, 15th, and 16th International Association for the Study of Pain conferences. Two independent reviewers screened titles and abstracts and cross-checked the extracted data.

**Results:**

The search retrieved 5546 articles. After full-text screening, 15 trials were included, which had randomised 1252 participants. There is moderate-quality evidence that in the short-term [WMD − 1.06, CI − 1.48 to − 0.64, *p* ≤ .01] and low-quality evidence that in the medium-term [WMD − 0.90, CI − 1.43 to − 0.37, *p* ≤ .01] postoperative period oral zolpidem 5/10 mg with other analgesic medicines reduced pain intensity compared to the same analgesic medicines alone.

There is low-quality evidence that melatonin was not effective on postoperative pain intensity compared to placebo. The results of benzodiazepines on pain intensity were mixed. The authors reported no significant adverse events.

**Conclusions:**

There is promising evidence that the hypnotic medicine zolpidem, adjuvant to other analgesics, is effective at achieving a minimally clinically important difference in pain intensity postoperatively. There is no consistent effect of melatonin or benzodiazepines on postoperative pain intensity. Readers should interpret these results with some caution due to the lack of data on safety, the small number of trials included in the pooled effects and their sample sizes.

**Systematic review registration:**

The protocol for this systematic review was registered with PROSPERO ID=CRD42015025327.

## Introduction

Postoperative pain is common; out of the estimated 48 million surgical procedures performed each year in the USA, 80% of patients report significant or severe pain in the postoperative period [1]. Acute postoperative pain is associated with decreased patient mobility, which can lead to an increased risk of complications such as deep vein thrombosis, pulmonary embolus and pneumonia [2]. Postoperative pain can also result in an extended hospital stay [2, 3], increase the risk of readmission [2, 3] and delay return to normal function and work [3]. Up to 50% of patients develop persistent postoperative pain [4], which is debilitating and can have legal and medico-economic consequences [5]. A consistent and strong predictor of persistent postoperative pain is intensity in the immediate postoperative period [6].

The evidence for the effectiveness of analgesic interventions for postoperative pain is typically low quality [7]. Only 4 out of 32 recommendations in the American Pain Society guideline for managing acute postoperative pain were supported by high-quality evidence [7]. Multimodal analgesia that includes opioids is recommended [7]. There is evidence that postoperative administration of opioids increases the risk of long-term opioid use following common surgical procedures such as total knee replacement (TKR) and laparoscopic cholecystectomy [8]. A recent Lancet series [9–11] highlighted the important role of non-opioid and opioid-sparing pharmacological interventions for the management of postoperative pain.

Following major operations, patients commonly report poor sleep quality [11]. Almost half (42%) of patients report unsatisfactory sleep after orthopaedic, vascular and general surgery [12]. Poor sleep quality is commonly managed with hypnotic medicines, including z-drugs, melatonin and benzodiazepines. These medicines may be provided postoperatively to improve sleep quality.

There is evidence that sleep quality and pain intensity have a bi-directional relationship [13]. For example, sleep quality was found to be associated with next day pain intensity, and daytime pain intensity was found to be associated with that night’s sleep quality for people with low back pain. These effects were independent of pain duration, depression and anxiety [13]. Given this relationship, it is possible that hypnotic medicines administered postoperatively to improve sleep quality may lead to reduced pain intensity and persistent postoperative pain. This effect has never been systematically evaluated. To our knowledge, this is the first systematic review to investigate the effect of hypnotic medicines on postoperative pain intensity.

The primary aim of this systematic review was to determine whether hypnotic medicines reduce postoperative pain intensity.

The key objectives were to determine whether hypnotic medicines:

- Reduce postoperative pain intensity,
- Reduce opioid consumption,
- Improve postoperative sleep outcomes.

We were also interested in whether any effects of hypnotic medicines on postoperative pain intensity are moderated by:

- The duration of symptoms,
- The type of surgery.

## Methods

### Protocol and registration

The study is reported in accordance with the PRISMA statement (Additional file 1) for the reporting of systematic reviews and meta-analyses [14], and the protocol was registered with PROSPERO ID=CRD42015025327 http://www.crd.york.ac.uk/PROSPERO/display_record.asp?ID=CRD42015025327.

### Eligibility criteria

Published randomised and quasi-randomised controlled trials (RCTs) from database inception, in any language, were considered for inclusion in the review. Inclusion criteria were defined using the PICO (Patients, Intervention, Control, Outcome) framework [14].

#### Patients

Trials that included adults older than 18 years of age who had undergone any surgery were eligible for the review. Trials that included a mixed sample of postoperative pain and other pain, such as low back pain, were excluded unless results for the postoperative sample were reported or could be obtained, separately.

#### Intervention

Trials that tested the effects of z-drugs ((e.g. zolpidem and zopiclone) a group of non-benzodiazepine hypnotics) [15], melatonin or benzodiazepine medicines, administered postoperatively as monotherapy or with analgesic interventions were eligible for inclusion. Trials were eligible if the hypnotic medicine was administered by oral, intravenous (IV), intramuscular (IM) or intranasal routes. Trials with an epidural or neuraxial mode of administration were excluded due to possible safety concerns. The most recent guideline for the management of postoperative pain states that the “neuraxial administration of benzodiazepines…in the treatment of postoperative pain is not recommended because of no clear benefit and insufficient evidence to determine safety” [7].

#### Comparison

Trials that compared a hypnotic medicine to (1) placebo, (2) analgesics (e.g. paracetamol, non-steroidal anti-inflammatory drugs (NSAIDs), opioids), (3) non-pharmacological modalities (e.g. cognitive-behavioral therapy-Insomnia (CBT-I), acupuncture, etc.) were included.

#### Outcomes

The primary outcome was pain intensity measured at any timepoint postoperatively. Trials that measured pain intensity using a valid and reliable assessment such as numeric rating scales (NRS) or visual analogue scales (VAS) were included.

Related outcomes included additional analgesia or narcotic consumption, measured as an exact dose or as the number of participants who requested additional analgesia.

Secondary outcomes are described in Additional file 2.

### Information sources

Sensitive search strategies were developed for Ovid MEDLINE, Ovid Embase, Cinahl, Psych info, Central and PubMed. Databases were searched from inception to July 2015 and updated in February 2020. Post hoc, the search was extended to include meta-Register of Controlled Trials and the Web of Science and the conference booklets for the 14th, 15th and 16th International Association for the Study of Pain conferences. The search strategy was adapted from a Cochrane review on postoperative pain and a separate review on hypnotic and sedating medicines [16, 17], modified to exclude search terms for sedating medicines. The MEDLINE search strategy is provided as Additional file 3.

### Study selection

Two independent reviewers screened titles and abstracts to identify trials that met the inclusion criteria (EO and either MH, CM, SG or CG). Disagreements between reviewers were resolved by discussion and consensus. Any remaining disagreements were resolved by consulting a third reviewer (JMcA). If the abstract was unclear, the article was retrieved, and two other independent reviewers reviewed the full text.

### Data collection process

One review author (EO) used a standardised report form to extract data from the eligible full-text trials. The extracted data were cross-checked by two reviewers (MH and JMcA).

### Data items

Extracted data included information on trial design and funding, recruitment source, patient characteristics, intervention, control, outcome measure assessed, duration of follow-up and results.

### Risk of bias in individual trials

One reviewer (EO) applied the Cochrane tool for assessing risk of bias [18] to each trial, and the score was reviewed by a second independent reviewer (MH, CM, SG or CG). A score of 1 was allocated if there was no or a low-risk of bias for each section, 0 if there was a high risk of bias, and “unclear” if the information was not clear from the manuscript. Disagreements between reviewers were resolved by discussion. The following domains were considered: random sequence generation (selection bias), allocation concealment (selection bias), blinding of participants, blinding of personnel/care providers (performance bias), blinding of outcome assessor (detection bias), incomplete outcome data (attrition bias), selective reporting (reporting bias), group similarity at baseline (selection bias), co-interventions (performance bias), compliance (performance bias), intention-to-treat-analysis attrition bias), timing of outcome assessments (detection bias), other bias which considered industry funding and ethics approval.

### Summary measures

Mean between-group differences and standard deviations for all outcomes were extracted from the manuscripts. For pain intensity, the primary outcome, if data were not reported on a 0–10 point scale, they were converted, where possible [14], i.e. outcome measures that used 1–4 scales were multiplied by 2.5, and 0–100 scales were divided by 10 [14]. Data were extracted for: immediate (up to 48 h postoperatively), short-term (48 h to 7 days postoperatively), medium-term (7–30 postoperatively) and long-term (greater than 1 month postoperatively) periods.

### Synthesis of results

#### Main effects of hypnotic medicines

The results from trials that were clinically homogenous were combined in a random-effects meta-analysis, and the weighted mean difference (WMD) was calculated using the RevMan review manager software, 5.3 [19]. Clinical homogeneity was determined by similarity of drug, mode of administration and control group.

#### Sub-group analyses

The different postoperative periods of analyses were combined into a single meta-regression analysis. We used random-effects meta-regression to investigate the relationship between pain and (1) hypnotic medicines over time, (2) control drug over time and (3) route of administration over time. A random effect for trial was included, as well as fixed effects of time and variable of interest (drug, control or route) and the interaction. For analyses involving a significant time*effect interaction, estimates of the effect size over time were obtained. Analyses were performed using the *metafor* package (version 3.4.3) in R [20].

We used the GRADE approach to assess the overall quality of the evidence for each outcome, as recommended in the *Cochrane Handbook for Systematic Reviews of Interventions* [18]. In line with this approach, we considered five factors for rating the quality of the evidence from high to no or very low-quality evidence. The five factors were (1) study design and risk of bias (downgraded if more than 25% of the participants were from studies with a high risk of bias), (2) inconsistency of results (downgraded if significant heterogeneity was present by visual inspection or if the *I*^2^ value was greater than 50%), (3) indirectness (generalisability of the findings; downgraded if more than 50% of the participants were outside the target group), (4) imprecision (downgraded if fewer than 400 participants were included in the comparison for continuous data and there were fewer than 300 events for dichotomous data) [21] and (5) other factors (e.g. reporting bias, publication bias).

##### High-quality evidence

There were consistent findings among at least 75% of RCTs with low-risk of bias, consistent, direct and precise data and no known or suspected publication biases. Further research is unlikely to change either the estimate or our confidence in the results.

##### Moderate-quality evidence

One of the domains is not met. Further research is likely to have an important impact on our confidence in the estimate of effect and may change the estimate.

##### Low-quality evidence

Two of the domains are not met. Further research is very likely to have an important impact on our confidence in the estimate of effect and is likely to change the estimate.

##### Very low-quality evidence

Three of the domains are not met. We are very uncertain about the results.

##### No evidence

No RCTs were identified that addressed this outcome.

#### Sensitivity analyses

A sensitivity analysis was planned to determine whether excluding trials of lower methodological quality or higher risk of bias affected the effects of the group comparisons. A post hoc sensitivity analysis was conducted to assess the effect of increasing the “immediate period” to 72 h postoperatively.

## Results

### Study flow

**Fig. 1:**
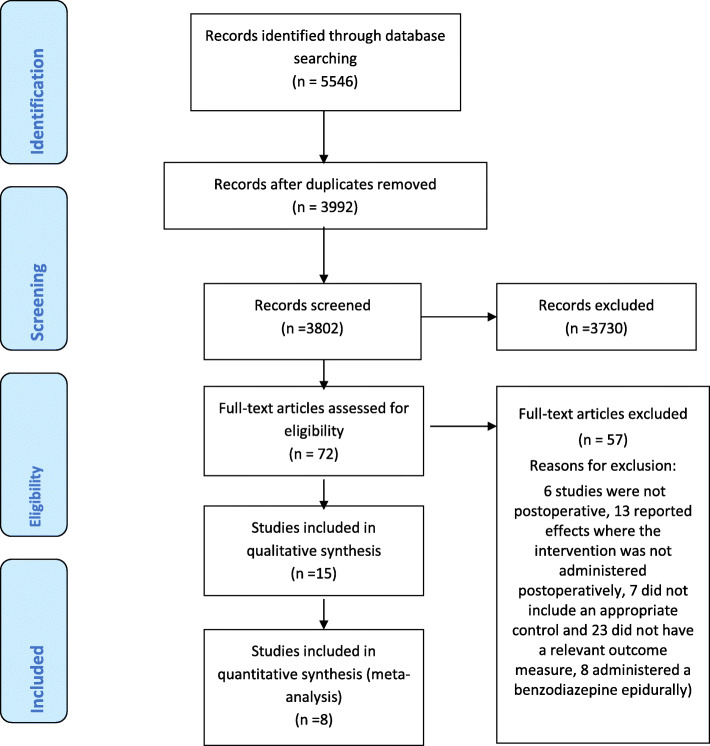
PRISMA flow chart

The search identified 5546 articles. Once duplicates and trials that did not meet inclusion criteria were removed, 72 articles remained. After full-text screening, 15 trials were included in the review (Fig. 1). Five authors were contacted to request additional data that was not reported in the trial report; one [22] provided the data, one replied that the additional data were no longer available [23] and 3 [24–26] did not respond after three attempts.

### Characteristics of included trials

The 15 included trials randomised a total of 1252 participants. Sample sizes of the trials ranged from 22 [24] to 250 [23]. Surgeries included gastrectomy or abdominal surgery (*n* = 4), spinal surgery (*n* = 2), orthopaedic lower limb surgery (*n* = 5), tracheostomy (*n* = 1), gynaecological surgery *n*=1, prostate resection (*n* = 1) and breast resection (*n* = 1). The trials investigated the effects of z-drugs with analgesic medicines vs the same analgesic medicines alone (*n* = 4), melatonin vs placebo (*n* = 2), benzodiazepines vs placebo (*n* = 3) and benzodiazepines with analgesic medicines vs the same analgesic medicines alone (*n* = 6). In 10 trials, the intervention was administered orally, IV in 3 trials, IM in one and via an intranasal spray in one trial.

Pain intensity was reported in 13 trials. Opioid consumption was measured in 7 trials. Two [22, 27] declared financial support from the pharmaceutical industry. Descriptive characteristics of each trial are provided in Table 1. Results for secondary outcomes are described in Table 2 and reported in greater detail in Additional file 2.

**Table 1:** Study characteristics

Author	Study design	Population	Intervention	Control	Outcomes
Bischoff et al. [23]	Randomised, placebo-controlled, double-blind trial. 2 arm trial. Sponsorship: none.	*N* = 250 (2 groups of 125); female (%), 57.6%/57.6%. Age 47 years (SD17)/47 years (SD15). Surgery: 32 different kinds of operation, most frequent being hernias, cholecystectomy, strumectomy, mastectomy, appendectomy and haemorrhoid operations	Benzodiazepine, lormetazepam, oral. Lormetazepam 2 mg PRN, nightly for 5 nights	Placebo, oral. Placebo PRN, nightly for 5 nights	1. Pain 100 mm VAS (completed in the am regarding degree of pain experienced during the night, every morning for 5 days)2. Analgesic use 3. Sleep quality 100 mm VAS -Total sleep duration (patients AX) -Sleep latency (min) -Awakenings (no.) -Feeling of freshness on awakening (100 mm VAS)
Blumenkopf [31]	Randomised, placebo-controlled, single-blind trial. 2 arm trial. Sponsorship: none.	*N* = 50 (intervention *n* = 21, control *n* = 29); Female (%), 36% overall. Age, 42 years (SD13) overall Surgery: spinal surgery.	Benzodiazepine in combination. Diazepam, oral. Diazepam 5 mg orally 3 times daily and baclofen 5 mg on day 1, 7.5 mg on day 2 and 10 mg on day 3 Access to promethazine hydrochloride 25 mg, plus meperidine hydrochloride 1 mg/kg q3H, IM as necessary for pain	Usual Care Access to promethazine hydrochloride 25 mg, plus meperidine hydrochloride 1 mg/kg q3H, IM as necessary for pain	1. Pain 0–10 NRS, measured day 1, 2, 3 post-surgery 2. Narcotic consumption
Bourne et al. [32]	Randomised, placebo-controlled, double-blind trial. 2 arm trial. Sponsorship: Sheffield Teaching Hospital Department of Pharmacy and Medicines Management.	*N* = 25 (intervention *n* = 13, placebo *n* = 12); female (%), 42%/67%. Age, 58.7 years (SD12.5) /69.9 years (SD12) Surgery: tracheostomy.	Melatonin, oral. 10 mg oral liquid melatonin, administered at 21:00 h for 4 nights	Placebo, oral. 10 mg liquid placebo, administered at 21:00 h for 4 nights	1. Richards Campbell Sleep Questionnaire -Sleep efficiency measured with EEG (and evaluated for agreement and reliability with actigraphy), measured at nights 3 and 4.
Egan et al. [27]	Randomised, placebo-controlled, double-blind trial. 2 arm trial. Sponsorship: none.	*N* = 40 (2 groups of 20); female (%), 100%/100%. Age, 42.9 years (SD12.2) /49.6 years (SD14.5). Surgery: abdominal total hysterectomy.	Benzodiazepine, midazolam IV. 0.125 mg midazolam. Access to PCA pump containing morphine 1 mg on demand (lockout period 8 min)	Placebo infusion.Access to PCA pump containing morphine 1 mg on demand (lockout period 8 min)	1. Pain 10 cm VAS, measured days 1 and 2 post surgery, between 13:00–15:00 h 2. Level of pain control (morphine) consumed
Gögenur et al. [33]	Randomised, placebo-controlled, double-blind trial. 2 arm trial. Sponsorship: none.	*N* = 136 (2 groups of 68); female (%), 68%/74%. Age, 44 years (SD13)/47 years(SD12) Surgery: laparoscopic cholecystectomy	Melatonin, oral. Three capsules of melatonin (each containing 5 mg) Over 3 nights Received 2.5–5.0 mg of Morphine IV. For post-op nausea .625 mg properidol and 1 mg ondansetron IV if necessary. Post discharge 8 mg lornoxicam 2 times a day at 20:00 h and 08:00 h for 4/7 and 5 mg ketobemidon as rescue analgesia	Placebo, oral. Three capsules of placebo (200 mg of lactose and 3 mg magnesium stearate) over 3 nights Received 2.5–5.0 mg of morphine IV. For post-op nausea .625 mg properidol and 1 mg ondansetron IV if necessary. Post discharge 8 mg lornoxicam 2 times a day at 20:00 h and 08:00 h for 4/7 and 5 mg ketobemidon as rescue analgesia	1. Pain 100 mm VAS, on waking and 20:00 h, on nights 1, 2 and3 post-surgery. 2. Self-reported sleep quality questionnaire 100 mm VAS, in am -Self report sleep quality via sleep diary, completed in am 3. Fatigue 100 mm VAS, on waking and at 20:00 h 4. General wellbeing on 100 mm VAS, on waking and at 20:00 h
Gong et al. [28]	Randomised, placebo-controlled, single-blind trial. 2 arm trial. Sponsorship: none.	*N* = 148 (2 groups of 74); female (%), 87%/ 83%. Age, 64.8 years (SD5.3)/66.1 yrs(SD5.9) Surgery: TKR	Non-benzodiazepine Zolpidem, oral. 5 mg zolpidem orally, at bed time for 14 nights Received tramadol 100 mg 3 times a day. Oxycodone and access to acetaminophen tablets (5 mg/325 mg) PRN	Placebo oral, at bed time, for 14 nights Received tramadol 100 mg 3 times a day. Oxycodone and access to acetaminophen tablets (5 mg/325 mg) PRN	1. Pain 0–10 VAS. Score as recall of previous night, measured at days 1, 3, 5, 7, 11 and14 post-surgery 2. Pain control consumption 3. Sleep Efficiency (PSG)
Hershberger and Milad [34].	Randomised, placebo-controlled, double-blind trial. 2 arm trial. Sponsorship: Institutional grant from Dept. of Obstetrics and Gynaecology, Northwestern University Feinberg School of Medicine.	*N* = 97 (intervention *n* = 49, placebo *n* = 48); female (%), 100%/100%. Age, 36 years (SD10)/35 years (SD6). Surgery: ambulatory gynaecological surgeries	Benzodiazepine, lorazepam IV. 1 mg lorazepam IV (single dose, 1 h post-surgery) Access to IV hydromorphone or intramuscular (IM) meperidine or IM fentanyl for pain control	Placebo, IV. 1 mg saline IV (single dose,1 h post-surgery) Access to IV hydromorphone or IM meperidine or IM fentanyl for pain control	1. Pain 0–10 VAS (number of pain score values recorded varied, but averaged 3, time varied, up to 8 h post op) 2. Narcotic consumption, mg of hydromorphone
Jacobsen et al. [29]	Randomised, placebo-controlled, double-blind trial. 2 arm trial. Sponsorship: none.	*N* = 100 (intervention *n* = 49, placebo *n* = 51); female (%) 100%/100%. Age, 49.9 years (SD10.5)/50.6 years (SD9.75). Surgery: breast resection, axillary node dissection or mastectomy.	Benzodiazepine, triazolam, oral. 0.125 mg triazolam 3 consecutive nights post op, with option to increase to 0.25 mg after 1 night Received morphine, meperidine, ibuprofen, acetaminophen and/or acetaminophen with codeine, oxycodone or propoxyphene	Placebo, oral. 0.125 mg placebo, permitted an increase to 0.25 mg Received morphine, meperidine, ibuprofen, acetaminophen and/or acetaminophen with codeine, oxycodone or propoxyphene	1. Sleep quality 100 mm VAS -Estimate TTS/sleep latency (min) -Number of awakenings -100 mm VAS difficulty falling asleep -100 mm VAS how rested they felt in the morning, every morning following 3 nights
Krenk et al. [24]	Randomised, placebo-controlled, double-blind trial. 2 arm trial. Sponsorship: Lundbeck Foundation Research Grant.	*N* = 22 (2 groups of 11); female (5%), 50%/70% overall. Age, 70.9 years (SD4.5)/70.5 years (SD5.5). Surgery: fast-track total hip replacement (THR) or total knee replacement (TKR)	Non-benzodiazepine Zolpidem, oral. Oral zolpidem 10 mg, administered 1 h prior to lights-out on day 1 post-op. Received celecoxib 200 mg, slow release acetaminophen 2 g twice daily and 300 mg gabapentin in the morning and 600 mg in the evening.	Placebo, oral. Received celecoxib 200 mg, slow release acetaminophen 2 g twice daily and 300 mg gabapentin in the morning and 600 mg in the evening.	1. Pain 0–100 mm VAS, morning of day 1 2.Narcotics consumed, mg of morphine 3. Subjective sleep quality 0–10 NRS -Objective sleep measures including TST, sleep period time (SPT), awake time (AT) 4. Fatigue 0–10 NRS
Nott et al. [26]	Randomised, placebo-controlled, double-blind trial. 4 arm trial. Sponsorship: none.	*N* = 80 (4 groups of 20); female (%): 0%/0%/0%/0%. Age, 71.7 years (SD9.8)/75.5 years (SD9.5)/71.2 years (SD8.2)/71.9 years (SD8.9). Surgery: uncomplicated transurethral prostatectomy	Group 1 Benzodiazepine Diazepam, oral. Oral diazepam 4 mg 3 times daily OR Group 2 Benzodiazepine in combination. Diazepam, oral. Oral diazepam and single epidural injection of 10 ml bupivacaine. All groups received IM pethidine 0.05–0.125 mg/kg with prochlorperazine 12.5 mg if needed or dextropropoxyphene 32.5 mg with paracetamol 325 mg	Group 3 Usual care OR Group 4 Usual care. Single epidural injection of 10 ml of bupivacaine All groups received IM pethidine 0.05–0.125 mg/kg with prochlorperazine 12.5 mg if needed or dextropropoxyphene 32.5 mg with paracetamol 325 mg	1. Pain (during irrigation) 0–2 scale (0 = no sensation, 1 = aware only, 2 = painful), within 2 days post-surgery 2. Narcotic consumption
Riediger et al. [35]	Randomised, placebo-controlled, double-blind trial. 2 arm trial. Sponsorship: University of Basel.	*N* = 24 (2 groups of 12); female (%), 63%/54% Age, 76 (SD11.75)/68 (SD10) Surgery: decompression for lumbar spine stenosis.	Benzodiazepine in combination. Midazolam, intranasal spray. 6 mg S-ketamine base and 0.5mcg chitosan-HCL/0.1 mL spray, with 0.75 mg midazolam intranasal unit-dose spray and 0.5mcg chitosan-HCL/0.1 mL per dose and placebo PCA. Received 1 g paracetamol orally every 6 h, access to IV metamizole 1 g rescue medication, 6 h lock out	Placebo 2 mg morphine IV PCA and placebo intranasal spray (saline 0.9% and 0.5mcg chitosan-HCL)Received 1 g paracetamol orally every 6 h Access to IV metamizole 1 g rescue medication, 6 h lock out	1. Pain 0–10 NRS, measured at 1, 2, 4, 24, 48 and 72 h post-surgery 2. Narcotic consumption
Sajedi et al. [30]	Randomised, placebo-controlled, double-blind trial. 3 arm trial [Intervention (I), intra-articular (IA), I intramuscular (IM), placebo (P)] Sponsorship: Research Dept. University of Medical Sciences, Isfahan.	*N* = 75 (3 groups of 25); female (%), 21%/20%/12%. Age, 26.9 years (SD5.4)/27.6 years (SD4.7)/27.5 years (SD5.1). Surgery: knee arthroscopy.	Group 1-IA (Benzodiazepine, midazolam intravenously (IV)). Midazolam (75mc/kg) and intra-articular isotonic saline (single dose) Access to inhalation of isoflurane (1–2%) and morphine (0.1 mg/kg) as additional anaesthesia OR Group 2-IV Benzodiazepine, midazolam intra-articular (IA). Midazolam (75mc/kg) and 10 cc IV isotonic saline (single dose) Access to inhalation of isoflurane (1–2%) and morphine (0.1 mg/kg)	Placebo IV and IA Access to inhalation of isoflurane (1–2%) and morphine (0.1 mg/kg)	1. Pain 0–10 VAS, at 2, 4, 8, 12 and 24 h 2.Analgesic consumption over 24 h
Singh et al. [36]	Randomised, placebo-controlled trial. 3 arm trial. Sponsorship: none.	*N* = 105 (3 groups of 35); female (%), 60%/63%/54% Age, 40 years (SD6.27)/38 years (SD7.11)/39 years (SD6.27). Surgery: upper abdominal surgery	Group 1 Benzodiazepine, diazepam, IM. Diazepam 10 mg IM (single dose) OR Group 2 Benzodiazepine, diazepam, IM. Diazepam 5 mg and morphine 5 mg, IM (single dose)	Morphine, IM. Morphine 10 mg IM (single dose)	1. Pain estimated by patient, 0–4 scale at 30, 60, 90 and120 min post-surgery -pain estimated by observer, 0–4 scale at 30, 60, 90 and 120 min post-surgery
Tashjian et al. [25]	Randomised, placebo-controlled, double-blind trial. 3 arm trial. Sponsorship: none.	*N* = 68 (intervention *n* = 24, control *n* = 24, placebo *n* = 19); female (%), 21%/26%/38% overall. Age, 49.2 years/47.5 years/47 years Surgery: knee arthroscopy.	Group 1 Non-benzodiazepine in combination Zolpidem, oral. 7 zolpidem tartrate tablets (10 mg) 40 hydrocodone/acetaminophen bi-tartrate tablets (7.5 mg/750 mg) for the first 7 postoperative days. Access to Ibuprofen 800 mg PRN	Group 2 Placebo, oral. Placebo and 40 hydrocodone/acetaminophen bi-tartrate tablets (7.5 mg/750 mg) Access to Ibuprofen 800 mg PRN OR Group 3 Usual care 40 hydrocodone/acetaminophen bi-tartrate tablets (7.5 mg/750 mg) Access to ibuprofen 800 mg PRN.	1. Pain 0-10 VAS measured morning and night for first 7 days 2. Pain control (hydrocodone/acetaminophen) consumed 3. Fatigue 0–10 VAS, measured morning and night for first 7 days
Tompkins et al. [22]	Randomised, placebo-controlled, double-blind trial. 2 arm trial. Sponsorship: Smith & Nephew.	*N* = 29 (intervention *n* = 13, control *n* = 16); female (%), 38%/56%. Age, 36.9 years/35.6 years. Surgery: primary arthroscopic ACL reconstruction.	Group 1 Non-benzodiazepine Zolpidem, oral. Zolpidem tartrate tablets (10 mg) taken for the first 7 post-operative days, taken nightly OR Group 2 Non-benzodiazepine in combination Zolpidem, oral. Oral zolpidem and 40 hydrocodone/acetaminophen bi-tartrate 7.5 mg, to be taken 1–2 tablet every 6 h PRN	Group 3 Placebo, oral OR Group 4 Placebo, oral. Placebo and 40 hydrocodone/acetaminophen bi-tartrate 7.5 mg, to be taken 1–2 tablet every 6 h PRN	1. Pain 0–100 VAS, measured in the morning and evening days 1–7 post-surgery 2. Narcotic consumed, vicodin (and ibuprofen) 3. Fatigue 0–100 VAS, measured in morning and evening days 1–7 post-surgery

**Table 2:** Secondary outcomes

	Time point	*n*	Outcome	Weighted mean difference (WMD)	Confidence interval	*p* value
**Sleep outcomes**						
Z-drugs in with analgesic medicines versus the same analgesic medicines alone	Immediate	20 (24)	Sleep quality (0–10 NRS)	− 1.60	− 2.91 to − 0.3	*p* ≤ .01
	Immediate	141 (28)	Sleep efficiency (PSG)	− 0.86	0.51 to 1.20	*p* ≤ .01
Melatonin versus placebo	Short-term	24 (35)	Sleep quality (Richards Campbell Questionnaire)	− 0.09	− 0.28 to 0.09	*p* = 0.32
	Short-term	121 (31)	Sleep quality (0–100 VAS)	− 0.09	− 0.27 to 0.45	*p* = 0.51
Benzodiazepines versus placebo	Immediate	344 (23) (30)	Sleep quality (0–100 VAS)	1.14	1.63 to 0.65	*p* < .01
	Short-term	250 (23)	Sleep quality (0–100 VAS)	0.60	0 to 1.20	*p* = 0.5
**Fatigue**						
Z-drugs with analgesic medicines versus the same analgesic medicines alone	Immediate	49 (22) (24)	Fatigue (0–10 NRS)	− 0.59	− 0.59 to 0.89	*p* = 0.56
	Short-term	70 (22) (25)	Fatigue (0–10 NRS)	0.29	− 0.21 to 0.78	*p* = 0.25
Melatonin versus placebo	Immediate	121 (31)	Fatigue (0–100 VAS)	− 0.40	− 1.16 to 0.36	*p* = 0.30
**Wellbeing**						
	Immediate	121 (31)	General wellbeing (0–100 VAS)	− 0.10	− 0.91 to 0.71	*p* = 0.81

### Study quality

A visual presentation of the risk of bias for each trial included is presented in Table 3. The most common risks of bias were did not provide data on group comparability at baseline (80%) and did not specify the method of allocation concealment (80%).

**Table 3:** Risk of bias for each trial included

	1	2	3	4	5	6	7	8	9	10	11	12	13
	Random sequence generation	Allocation concealment	Blinding of participants	Blinding of personnel/care providers	Blinding of outcome assessor	Incomplete outcome data	Selective reporting	Group similarity at baseline	Co-interventions	Compliance	Intention-to-treat analysis	Timing of outcome assessments	Other Bias
Bischoff et al. [23]	Unclear	Unclear	Unclear	Unclear	Unclear	0	Unclear	Unclear	1	1	Unclear	1	Unclear
Blumenkopf [31]	0	Unclear	1	0	0	1	1	Unclear	1	Unclear	1	1	Unclear
Bourne et al. [32]	Unclear	1	1	Unclear	Unclear	1	1	1	1	1	1	1	1
Egan et al. [27]	Unclear	Unclear	1	1	1	1	Unclear	1	1	1	1	1	Unclear
Gögenur et al. [33]	1	1	1	1	1	1	1	Unclear	1	1	1	1	1
Gong et al. [28]	1	Unclear	1	1	1	1	1	1	1	1	1	1	1
Hershberger and Milad [34]	1	1	1	1	1	1	1	Unclear	1	1	1	1	Unclear
Jacobsen et al. [29]	Unclear	1	1	1	1	1	1	1	Unclear	1	0	1	0
Krenk et al. [24]	1	1	1	1	1	1	1	1	1	1	0	1	1
Nott et al. [26]	Unclear	Unclear	Unclear	Unclear	1	1	0	Unclear	1	1	Unclear	1	1
Riediger et al. [35]	1	Unclear	1	1	1	1	1	1	1	1	1	1	1
Sajedi et al. [30]	1	1	1	1	1	1	1	Unclear	1	1	1	1	1
Singh et al. [36]	Unclear	Unclear	Unclear	Unclear	1	1	Unclear	Unclear	1	1	1	1	Unclear
Tashjian et al. [25]	1	1	1	1	1	1	1	1	1	Unclear	1	1	Unclear
Tompkins et al. [13]	Unclear	1	1	1	1	1	1	Unclear	1	Unclear	1	1	0

### Other potential sources of bias

#### Publication bias

We did not assess publication bias with funnel plots because too few studies were included in the meta-analysis.

### Synthesis of results

Data from 8 trials [22–25, 27–30] were combined in a meta-analysis.

The results from 7 trials [26, 31–36] were synthesised narratively due to heterogeneity of the type of intervention (i.e. different combinations of hypnotic medicines and other pain medicines), route of administration (i.e. oral, IV, IM), comparison group (i.e. the same analgesic medicines or different analgesic medicines or placebo) or timing of outcomes assessment (immediately, short-term, medium-term, long-term).

### The main effect of hypnotic medicines on pain intensity

#### The effect of z-drugs with analgesic medicines versus the same analgesic medicines alone (Fig. 2)

There is moderate-quality evidence (downgraded due to imprecision) that in the immediate postoperative period (2 trials, *n* = 161 [24, 28]) the effect of oral zolpidem 5/10 mg (taken at night) with other analgesic medicines compared to the same analgesic medicines alone on pain intensity was not significant [WMD − 0.25, CI − 0.81 to 0.31, *p* = 0.38].

**Fig. 2:**
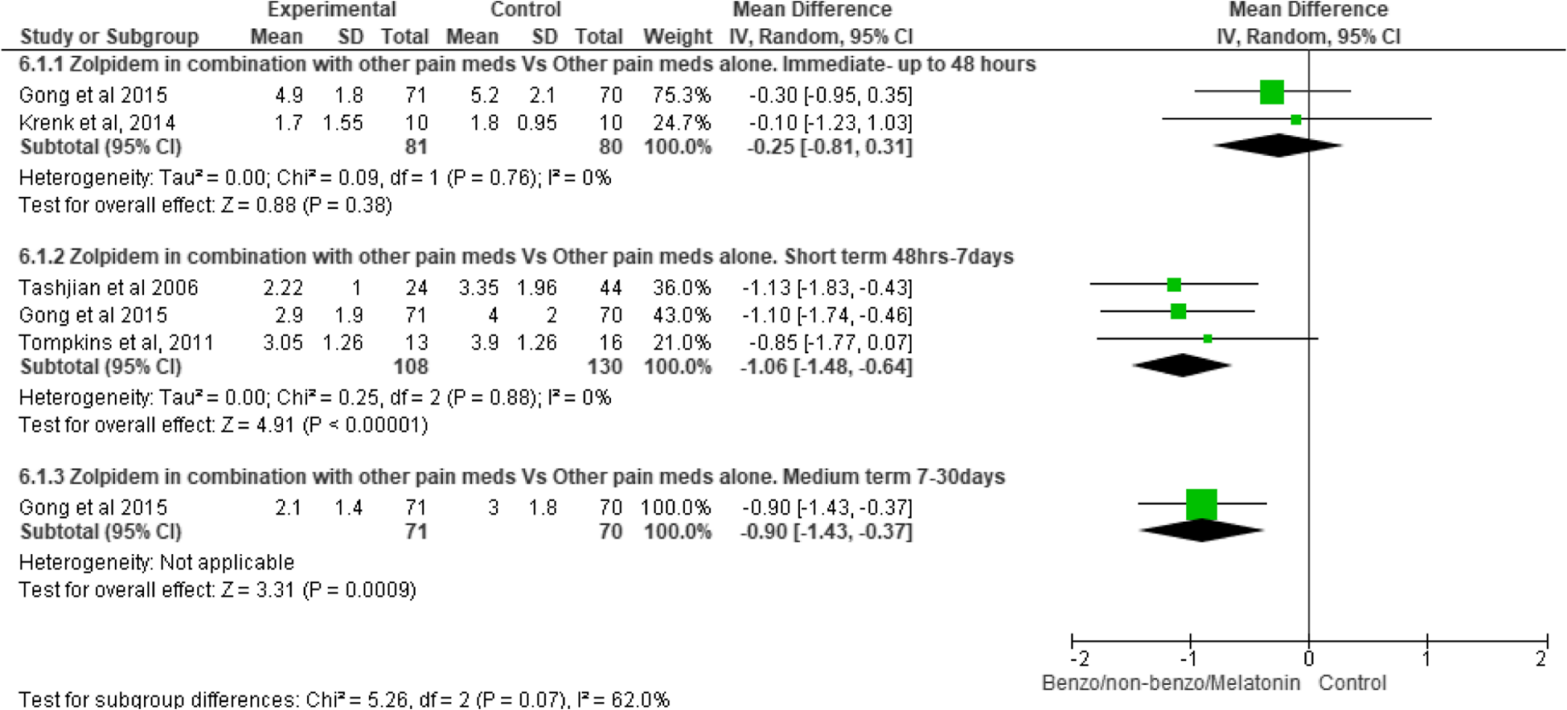
Z-drugs with analgesic medicine versus the same analgesic medicines alone

There is moderate-quality evidence (downgraded due to imprecision) that in the short-term postoperative period (3 trials, *n* = 238 [22, 25, 28]) the effect of oral zolpidem (taken at night for seven nights) with other analgesic medicines significantly decreased pain intensity compared to the same analgesic medicines alone [WMD − 1.06, CI − 1.48 to − 0.64, *p* ≤ .01].

There is low-quality evidence (downgraded due to imprecision and inconsistency) that in the medium-term postoperative period (1 trial, *n* = 141 [28]) the effect of oral zolpidem 5 mg (taken at night for 14 nights) with other analgesic medicines significantly decreased pain intensity compared to the same analgesic medicines alone [WMD − 0.90, CI − 1.43 to − 0.37, *p* ≤ .01].

#### Melatonin versus placebo

There is low-quality evidence (downgraded due to imprecision and inconsistency) that in the short-term postoperative period (1 trial, *n* = 121 [33]) the effect of oral melatonin 5 mg (taken at night for three nights) on pain intensity compared to placebo was not significant [WMD 0.10, CI − 0.61 to 0.81, *p* = 0.78].

#### Benzodiazepines versus placebo (Fig. 3)

There is moderate-quality evidence (downgraded due to imprecision) that in the immediate post-operative period (2 trials, *n* = 89 [27, 30]) the effect of an infusion of midazolam (0.125 mg/kg or 0.075/kg) on pain intensity compared to placebo was not significant [WMD − 0.00, CI − 0.29 to 0.29, *p* = 1.00].

**Fig. 3:**
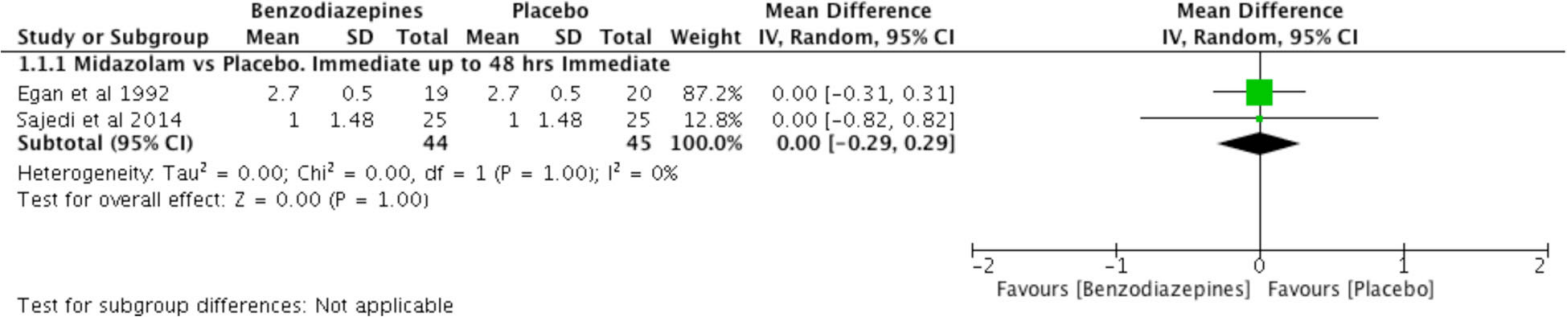
Benzodiazepines versus placebo

There is low-quality evidence (downgraded due to imprecision and inconsistency) that in the immediate postoperative period (1 trial, *n* = 97 [34]) the effect of intravenous lorazepam 1 mg (4 times a day) was significantly less effective on pain intensity compared to placebo [WMD 1.00, CI 0.22 to 1.78, *p* ≤ .01]. This trial was not included in the meta-analysis due to the use of a different benzodiazepine.

#### Benzodiazepines versus other analgesic medicines

There is very low-quality evidence (downgraded due to high risk of bias, imprecision and inconsistency) that in the immediate postoperative period (1 trial, *n* = 105 [36]) the effect of intramuscular diazepam 10 mg (4 times a day) on pain intensity compared to intramuscular morphine 10 mg (4 times a day) was not significant [WMD 2.00, CI 1.53 to 2.47, p ≤ .01].

#### Benzodiazepines with other analgesic medicines versus the same analgesic medicines alone (not meta-analysed due to variability in the timing of outcomes and the control groups)

There is very low-quality evidence (downgraded due to high risk of bias, imprecision and inconsistency) that in the immediate postoperative period (1 trial, *n* = 80 [26]) the effect of oral diazepam, 4 mg (3 times a day) with intramuscular pethidine and prochlorperazine, or oral dextropropoxyphene and paracetamol was significantly more effective on pain intensity compared to the same analgesic medicines alone [WMD − 4.0, CI − 5.55 to − 2.45, *p* ≤ .01].

There is very low-quality evidence (downgraded due to high risk of bias, imprecision and inconsistency) that in the short-term postoperative period (1 trial, *n* = 50 [31]) the effect of oral diazepam 5 mg (3 times a day, for 3 days) with oral promethazine, pethidine and baclofen on pain intensity compared with promethazine and pethidine was not significant [WMD − 0.14, CI − 0.84 to 1.21, *p* = 0.21].

There is low-quality evidence (downgraded due to imprecision and inconsistency) that in both the immediate and short-term postoperative period (1 trial, *n* = 22 [35]) the effect of intranasal midazolam 0.75 mg with 6 mg S-Ketamine base and 0.5 mcg chitosan-HCL/0.1 mL spray, and placebo patient-controlled analgesia (PCA) on pain intensity compared with 2 mg morphine IV PCA and placebo intranasal spray (saline 0.9% and 0.5mcg chitosan-HCL) was not significant [immediate WMD − 0.70, CI − 1.59 to 0.19, *p* = 0.12 and short-term WMD − 0.65, CI − 1.35 to 0.05, *p* = 0.07]

### Effects of hypnotic medicines on opioid consumption

#### Z-drugs with analgesic medicines versus the same analgesic medicines alone (not meta-analysed due to variability in the timing of outcomes and the control groups)

There is low-quality evidence (downgraded due to imprecision and inconsistency) that in the immediate postoperative period (1 trial, *n* = 20 [24]) oral zolpidem 5/10 mg (taken at night) with other analgesic medicines was not associated with opioid consumption (measured as the number of milligrammes of morphine consumed) compared to the same analgesic medicines alone [WMD − 1.00, CI − 22.98 to 20.98, *p* = − 0.93].

There is moderate-quality evidence (downgraded due to imprecision) that in the short-term postoperative period (2 trials, *n* = 97 [22, 25]), the effect of oral zolpidem (taken at night for seven nights) with other analgesic medicines significantly decreased opioid consumption (measured hydrocodone/acetaminophen bitartrate consumed) compared to the same analgesics medicines alone [WMD − 3.04, CI − 5.73 to − 0.35, *p* = 0.03].

There is low-quality evidence (downgraded due to imprecision and inconsistency) that in the medium-term postoperative period (1 trial, *n* = 141 [28]) the effect of oral zolpidem 5 mg (at night for 14 nights) with other analgesic medicines significantly decreased opioid consumption (measured as the number of milligrammes of morphine consumed) compared to the same analgesic medicines [WMD − 116.90, CI − 131.88 to − 101.92, *p* < .01].

#### Benzodiazepines versus placebo

There is moderate-quality evidence (downgraded due to imprecision) that in the immediate postoperative period (2 trials, *n* = 89 [27, 30]) the effect of an infusion of midazolam (0.125 mg/kg or 0.075/kg) was not associated with opioid consumption (measured as the number of milligrammes of morphine consumed) compared to placebo [WMD − 20.71, CI − 49.63 to 8.22, *p* = 0.16].

The following trial was not added to the meta-analysis as it reported a different combination of hypnotic medicines.

There is very low-quality evidence (downgraded due to high risk of bias, imprecision and inconsistency) that in the immediate postoperative period (1 trial, *n* = 97 [36]) the effect of intravenous lorazepam 1 mg (4 times a day) was not associated with opioid consumption (measured as the number of milligrammes of fentanyl consumed) compared to placebo [WMD 0.20, CI − 0.10 to 0.50, *p* = 0.20].

### Sub-group analyses

#### Effect of hypnotic medicines on pain intensity over time (hypnotic medicine by time interaction)

**Fig. 4:**
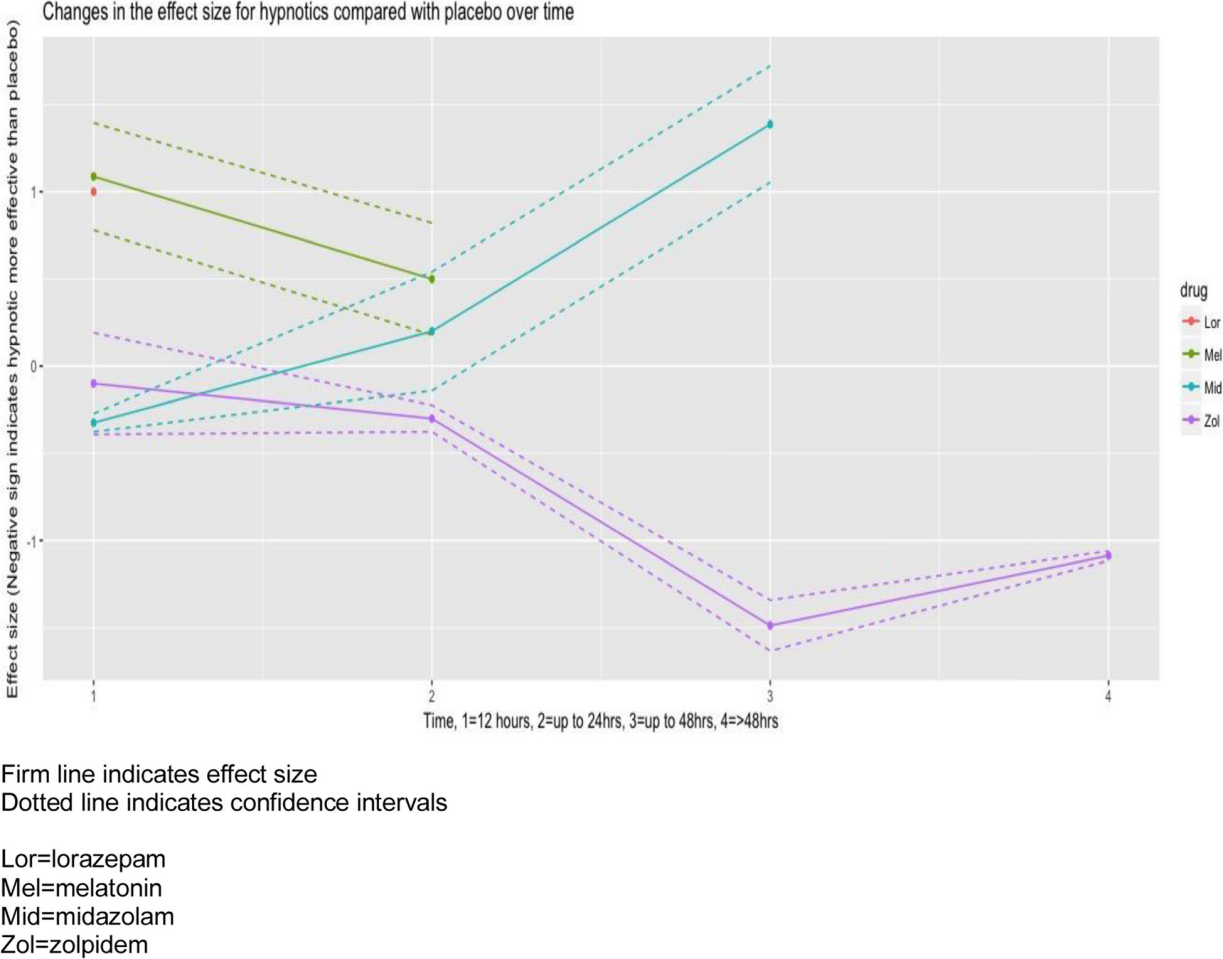
Effectiveness of hypnotics over time

The effect of each hypnotic medicine on pain intensity over time is presented in Table 4 and Fig. 4.

**Table 4:** Effectiveness of hypnotic medicines over time

	< 12 h	12–24 h	24–36 h	> 48 h
Zolpidem	WMD − 0.1 CI − 0.39 to 0.19 *p* value < 0.01	WMD − 0.3 CI − 0.38 to − 0.22 *p* value < 0.01	WMD − 1.49 CI − 1.63 to − 1.34 *p* value < 0.01	WMD − 1.09 CI − 1.12 to − 1.06 *p* value < 0.01
Melatonin	WMD 1.09 CI 0.77 to 1.40 *p* value < 0.01	WMD 0.50 CI 0.18 to 0.82 *p* value < 0.01	n/a	n/a
Midazolam	WMD − 0.32 CI − 0.38 to − 0.27 *p* value < 0.01	WMD 0.20 CI − 0.14 to 0.54 *p* value < 0.01	WMD 1.39 CI 1.05 to 1.72 *p* value < 0.01	n/a
Lorazepam	WMD 1.00 CI 0.88 to 1.12 *p* value < 0.01	n/a	n/a	n/a

*WMD* weighted mean difference, *CI* confidence interval, *n/a* no measure for this time point

The effect of zolpidem on pain intensity achieves a clinically important difference of 1-point on a 10-point scale [37] from 24 h postoperatively. This clinically important difference in effect size of zolpidem compared to placebo is maintained at 7 days [WMD − 1.09, CI − 1.12 to − 1.06, *p* < 0.01].

Melatonin is significantly less effective at reducing pain intensity than placebo at both 12 h [WMD 1.09, CI 0.77 to 1.40, *p* < 0.01] and 24 h [WMD 0.5, CI 0.18 to 0.82, *p* < 0.01].

Midazolam was more effective than placebo at reducing pain intensity up to 12 h postoperatively [WMD − 0.32, CI − 0.38 to 0.27, *p* < 0.01]. Midazolam is less effective than placebo at both 24 h [WMD 0.20, CI 0.18 to 0.82, *p* < 0.01] and at 36 h post-operatively [WMD1.39, CI 1.05 to 1.72, *p* < 0.01]. Lorazepam was less effective than placebo at reducing pain intensity over time [WMD 1.00, CI 0.88 to 1.12, *p* < 0.01].

#### The effect of type of comparison medicine on the relationship between hypnotic medicines and pain intensity

The effect of hypnotic medicines on pain intensity varied depending on the type of comparison medicine. Overall, the effect of hypnotic medicines compared to placebo on pain intensity was not significant [WMD = − 0.29, CI − 0.98 to 0.39, *p* = 0.19]. Hypnotic medicines were not more effective at decreasing pain intensity compared to morphine [WMD = 0.68, CI − 0.70 to 2.05, *p* = 0.33]. Hypnotic medicines with analgesic medicines were significantly more effective at decreasing pain intensity than the same analgesics alone [WMD = − 2.14, CI − 3.52 to − 0.76, *p* < 0.01].

#### The effect of route of administration on the relationship between hypnotic medicine and pain intensity

Hypnotic medicines were significantly more effective at decreasing pain intensity when delivered orally [WMD = − 0.85, CI − 1.58 to − 0.12, *p* = 0.02]. Hypnotics were not more effective at decreasing pain intensity when delivered via an infusion [WMD = 0.23, CI − 0.34 to 0.81, *p* = 0.43].

The trials in which the hypnotic medicines were delivered intranasally or intramuscularly were not compared to a placebo so were not included in the meta-regression.

There were insufficient data to perform subgroup analysis to determine whether the effects on pain intensity were moderated by the duration of symptoms or type of surgery.

### Sensitivity analysis

We were unable to conduct a sensitivity analysis to determine whether excluding trials of lower methodological quality or higher risk of bias affected the effects of the group comparisons due to the small number of trials. With one exception [23], the trials included in the meta-analysis had more endorsed quality items than those not included (Table 2).

A post hoc sensitivity analysis was conducted to assess the effect of increasing the “immediate period” to 72 h postoperatively. Only one trial had additional data for this time period [28]. The inclusion of these data did not change the results. There was no effect of non-benzodiazepines with other analgesics on pain intensity up to 72 h postoperatively [WMD − 0.71, CI − 1.67 to 0.24, *p* = 0.14].

## Discussion

### Summary of evidence

This systematic review found moderate-quality evidence that the hypnotic medicine zolpidem, a z-drug, administered in the postoperative period, has an analgesic effect in the short- and medium-term. The effect size is greatest at 36 h postoperatively, and a clinically important difference of 1-point on a 10-point scale [37] is maintained 1 week postoperatively. This effect is apparent when the hypnotic medicine is co-administered with analgesic medicines and not when administered as monotherapy. This finding raises the possibility that z-drugs could play an important adjuvant role for postoperative pain management.

Melatonin was not effective at reducing postoperative pain compared to placebo. The results for the effect of benzodiazepines with analgesic medicines on pain intensity are mixed. While one [26] trial reported a significant decrease in pain intensity immediately postoperatively, a second showed no effect [35].

Although we were unable to determine whether the effects of hypnotic medicines on pain intensity are moderated by the duration of symptoms or type of surgery due to a limited number of studies, we found that the effect of hypnotic medicines was increased when delivered orally. Hypnotic medicines have an adjunctive role; our results show they are effective at decreasing pain intensity when combined with other analgesic medicines. When participants who only received hypnotics were compared with participants only receiving placebo, there was not a significant change in pain intensity. Similarly, when participants only received hypnotics were compared with participants receiving morphine, there was not a significant change in pain intensity.

The strengths of this study are that, to our knowledge, this is the first systematic review to investigate the effect of hypnotic medicines on pain intensity, sleep quality and opioid consumption postoperatively. We supplemented the analysis by conducting a mixed-methods meta-regression to investigate whether the different medicine classes (z-drugs and benzodiazepines) had different effects on pain intensity over time (Fig. 4).

The findings can be used to guide future research. The current guidelines on postoperative pain management recommend “clinicians offer multimodal analgesia, or the use of a variety of analgesic medicines and techniques combined with non-pharmacological interventions, for the treatment of postoperative pain in children and adults” [7]. Emphasis is placed on the importance of minimising opioid therapy as the guideline recommends clinicians incorporate routine non-opioid analgesics. Alternatives to opioids for multimodal pain management are necessary to further decrease the opioid dependence and prevent chronic opioid use.

#### Effect on postoperative pain intensity

We found a weighted mean between-group difference of 1.5 on an 11-point scale for pain intensity when zolpidem with analgesics was compared to placebo zolpidem and the same analgesics at 36 h postoperatively. At 7 days postoperatively, this effect was 1.1. Although the absolute effects are modest, the reduction in pain intensity is in addition to that obtained from the other analgesics, suggesting a possible adjuvant role in postoperative pain management.

#### Effect on opioid consumption

The evidence for opioid-sparing effects of zolpidem was mixed. It is noteworthy that the larger trial, of higher quality [28], found that the intervention group, who were receiving zolpidem, required significantly less morphine over the course of the 14-day investigation period compared to the placebo group. These preliminary findings may have identified a promising alternative analgesic option with opioid-sparing effects.

#### Effect on postoperative sleep outcomes

Improved sleep quality was highlighted as an important outcome in a report on research gaps in clinical guidelines for the management of acute postoperative pain [38]. We investigated whether the use of a hypnotic drug administered postoperatively improved sleep outcomes. While we were unable to pool trials to determine the effect of z-drugs on sleep outcomes, our results suggest that benzodiazepines are more effective than placebo at improving sleep outcomes postoperatively.

This systematic review raises numerous questions for future research. Future research should consider other factors that influence pain intensity and sleep outcomes in the postoperative period, including anxiety, nursing interventions and environmental disturbances. Le Guen et al. [39], for instance, found that sleep quality was significantly improved postoperatively by the use of a simple intervention, earplugs and eye masks, which significantly decreased the total consumption of morphine.

Secondly, the benefits of melatonin may be underestimated in this trial due to the relatively small doses administered. Enhanced effects may be observed at higher dosages. Third, zolpidem’s hypnotic effect is achieved by increasing GABA activity at the GABA-A receptor [40]. Z drugs unselectively bind to GABA-A subunits 1, 2, 3 and 5 [41, 42]. GABA-A subunit 1 is known to be strongly associated with sedation [43]. There is promising evidence for the usefulness of hypnotic medications as part of an opioid-free multimodal balanced anaesthesia strategy to achieve sedation necessary for major surgery [44]. It is not known whether sedative hypnotics have an opioid sparing effect, though as both medicines are CNS depressant drugs, factors other than analgesia may be in play. These sedative effects of z-drugs should be investigated to maximise the parallels between achieving sedation and reducing pain intensity.

There are some limitations in our review that have reduced the strength of our conclusions. Firstly, sample sizes of the included trials were typically small; trials were conducted on samples of 22–250 participants; 9 trials included less than 100 participants. In our review, the risk of bias assessment suggested that over a quarter (4 out of 15) of the included trials were at high or unclear risk of bias. It is noteworthy that the most common potential sources of bias were that trials did not provide data on group comparability at baseline (53%) and failed to specify the method of allocation concealment (47%) or randomisation (47%). Trials of zolpidem were typically of higher quality (Table 2).

No medicine is without hazards. Z-drugs are associated with an increased falls risk in older adults [36, 45]. The trials included in our systematic review did not find a significant difference in the rate or severity of adverse events between the intervention or control groups. Six of the included trials did not report on adverse events, and 9 reported non-significant adverse events. The most commonly reported adverse events included headache [32], nystagmus [35] and urinary retention [31]. Adverse events are known to be under-reported in clinical trials. Even if adverse events were adequately reported, we acknowledge that RCTs traditionally have limited capacity to evaluate the safety of a medicine [46]. As the trials included in this review have small sample sizes, short follow-ups and restrictive inclusion criteria, they are a poor method for assessing safety. Consequently, side effects may be under-reported [46]. This makes it difficult to conclusively weigh the benefits of these interventions against harms.

There are some concerns that hypnotics delay recovery and discharge postoperatively [47]. We were unable to evaluate this effect as none of the included trials reported time to discharge as an outcome.

The trials we included that investigated the effect of zolpidem on pain intensity postoperatively were conducted on patients undergoing orthopaedic surgery. It is not clear whether the type of surgery or the patient population is important when investigating the effect of the hypnotic medications on pain intensity.

Finally, we conducted a meta-regression to investigate the efficacy of hypnotics in relation to how they were administered. The results are not clinically useful as z-drugs are routinely administered orally only.

### Clinical implications

We found that zolpidem, in combination with other analgesics, significantly improved pain intensity and sleep quality postoperatively, with modest effects. We found a consistent decrease in pain intensity of more than 1 point on a 10-point scale when zolpidem with analgesic medicines was compared to a placebo between 24 h and 7 days postoperatively. Individually, the trials that investigated the effect of zolpidem on pain intensity postoperatively were of low-risk of bias, but when the evidence is taken as a whole, the strength of the evidence is reduced. This is due to the trials eligible for inclusion having small sample sizes. This is an exploratory study that adds to the body of literature as the first systematic review to investigate the effect of hypnotic medicines on pain intensity, sleep quality and opioid consumption postoperatively. It supports recent evidence that perioperative addition of melatonin or zolpidem may improve postoperative pain control [48]. Although the absolute effects are modest, the reduction in pain intensity, improvement in sleep quality and reduction in opioid consumption suggests that these medicines may have an adjuvant role in postoperative pain management.

## Conclusion

This study has identified areas for future research, including the opioid-sparing effects of hypnotics and the potential benefit of addressing sleep quality to improve pain intensity postoperatively. The current results should be interpreted with caution due to lack of data on safety, a small number of trials included in the pooled effects and their sample sizes. In general, future studies should include a greater number of participants, a robust safety protocol and record time to discharge. Specifically, the effect of zolpidem on pain intensity postoperatively should be investigated as an adjunct to simple analgesic medicines *only* to understand the opioid-sparing potential of zolpidem.

## Supplementary information

**Additional file 1.** PRISMA Checklist

**Additional file 2.** Secondary Outcomes

**Additional file 3.** Search Terms

## Data Availability

The datasets used and/or analysed during the current study are available from the corresponding author on reasonable request.

## Abbreviations

CBT-I: Cognitive Behavioural Therapy-Insomnia
GABA: Gamma aminobutyric acid
IM: Intramuscular
IV: Intravenous
NRS: Numeric Rating Scale
NSAIDS: Non-steroidal anti-inflammatory drugs
PCA: Patient-controlled analgesia
RCT: Randomised controlled trial
TKR: Total knee replacement
VAS: Visual analogue scale
WMD: Weighted mean difference
Z-drugs: A group of non-benzodiazepine hypnotics
