# Supplementary material for: Zolpidem reduces pain intensity postoperatively: a systematic review and meta-analysis of the effect of hypnotic medicines on post-operative pain intensity
Source: Syst Rev. 2020 Sep 3;9:206. doi: 10.1186/s13643-020-01458-8 (PMC7472584; doi:10.1186/s13643-020-01458-8)
Supplement: Supplementary file 2 — Additional file 2. Secondary Outcomes [file 13643_2020_1458_MOESM2_ESM.docx]

**Appendix 2- Secondary Outcomes**

**Methods**

*Secondary outcomes*

The following secondary outcomes were assessed by a validated questionnaire;

2. Sleep quality or sleep efficiency3. Disability,

4. Fatigue,

5. Quality of life and general health,

6. Mental wellbeing/distress.

**Results**

Sleep outcomes were reported in 6 trials, and both pain intensity and sleep outcomes were reported in 4 trials. (24) (25) (28) (31) Sleep quality was measured on an 11-point sleep quality NRS, a 0-100 point VAS or by the Richards Campbell sleep questionnaire. Disability was reported in 0 trials. Fatigue was reported in 3 trials (22) (24) (31), measured on an 11-point sleep quality NRS or a 0-100 point VAS. General wellbeing was reported in 1 trial (31) measured on an 0-100-point VAS.

Effects of hypnotic medicines on sleep outcomes

*Z-drugs in with analgesic medicines versus the same analgesic medicines alone*

The following trials were not combined in a meta-analysis as the outcomes differed across trials.

In the immediate postoperative period (1 trial, n=20 (24)) the effect of effect of oral zolpidem 10mg (taken at night) with other analgesic medicines significantly improved sleep quality compared to the same analgesic medicines alone [WMD -1.60, CI -2.91 to -0.3, p≤.01].

In the immediate postoperative period (1 trial, n=141 (28)) the effect of effect of oral zolpidem 5 mg (taken at night for 14 nights) with other analgesic medicines significantly improved sleep efficiency, measured with polysomnography (PSG) compared to the same analgesic medicines alone [WMD 0.86, CI 0.51 to 1.20, p≤.01].

*Melatonin versus placebo* (not meta-analysed due to variability in outcome measures used)

In the short-term postoperative period (1 trial, n=24 (35)) the effect of effect of oral melatonin 10 mg (taken at night for four nights) on sleep quality (measured with Richards Campbell sleep questionnaire) compared with placebo was not significant [WMD -0.09, CI -0.28 to 0.09, p=0.32].

In the short-term postoperative period (1 trial, n=121 (31)) the effect of effect of oral melatonin 10 mg (taken at night for four nights) on sleep quality (measured with a 0-100 point sleep quality VAS) compared with placebo was not significant [WMD 0.09, CI -0.27 to 0.45, p=0.51].

*Benzodiazepines versus placebo*

In the immediate postoperative period, (2 trials, n=344 (23) (30)) the effect of oral lormetazepam 2mg (prn for 5 nights) or oral triazolam 0.125mg (for 3 nights) significantly improved sleep quality (measured with a 0-100-point sleep quality VAS) compared to placebo [WMD 1.14, CI 1.63 to 0.65, p<.01].

The following trial was not added to the meta-analysis as the timing of outcome measures differed across trials.

In the short-term postoperative period, (1 trial, n=250 (23)) the effect of oral lormetazepam 2mg (PRN for five nights) significantly improved sleep quality (measured with a 0-100-point sleep quality VAS) compared to placebo [WMD 0.60, CI 0 to 1.20, p=.05].

Effects of hypnotic medicines on fatigue

*Z-drugs with analgesic medicines versus the same analgesic medicines alone*

In the immediate postoperative period, (2 trials, n=49 (22) (24)) the effect of oral zolpidem 10mg (taken at night) with other analgesic medicines on fatigue (measured with an 11-point NRS) compared to placebo was not significant [WMD -0.59, CI -0.59 to 0.89, p=0.56].

In the short-term postoperative period, (2 trials, n=70 (22) (25)) the effect of oral zolpidem 10mg (taken at night) with other analgesic medicines on fatigue (measured with an 11-point NRS) compared to placebo was not significant [WMD 0.29, CI -0.21 to 0.78, p=0.25].

*Melatonin versus placebo*

Gögenur et al. (31) (n=121) investigated the effect of oral melatonin on fatigue. They measured fatigue, (measured with a 0-100-point VAS), immediately postoperatively. The effect of melatonin, 5mg (x3 nights), on fatigue [WMD -0.40, CI-1.16 to 0.36 p=0.30] was not significant.

The effect of hypnotic medicines on general wellbeing

*Melatonin versus placebo*

In the immediate postoperative period (1 trial, n=121 (31)) the effect of oral melatonin 10 mg (taken at night for four nights) on general wellbeing (measured with a 0-100 point VAS) compared with placebo was not significant [WMD -0.10, CI-0.91 to 0.71) p=0.81]
